# The Trophic Significance of the Indo-Pacific Humpback Dolphin, *Sousa chinensis*, in Western Taiwan

**DOI:** 10.1371/journal.pone.0165283

**Published:** 2016-10-25

**Authors:** Ching-Wen Pan, Meng-Hsien Chen, Lien-Siang Chou, Hsing-Juh Lin

**Affiliations:** 1 Department of Life Sciences and Research Center for Global Change Biology, National Chung Hsing University, Taichung 402, Taiwan; 2 Department of Oceanography and Asia-Pacific Ocean Research Center, National Sun Yat-sen University, Kaohsiung 804, Taiwan; 3 Department of Biomedical Science and Environmental Biology, Kaohsiung Medical University, Kaohsiung 807, Taiwan; 4 Institute of Ecology and Evolutionary Biology, National Taiwan University, Taipei 106, Taiwan; 5 Biodiversity Research Center, Academia Sinica, Taipei 115, Taiwan; Institute of Deep-sea Science and Engineering, Chinese Academy of Sciences, CHINA

## Abstract

Indo-Pacific humpback dolphins (*Sousa chinensis*) have attracted considerable attention due to their critically endangered status and related conservation issues, but their trophic relationships and ecological significance in coastal ecosystems are poorly understood. For instance, this species is noticeably more abundant in the Xin-Huwei River Estuary (Ex) of Western Taiwan than in the nearby Zhuoshui River Estuary (Ez), though it is unclear why the distribution shows such partitioning. To explore this topic, we conducted field surveys seasonally for two years from 2012 to 2013 and constructed Ecopath models of Ex, Ez, and an offshore site (Dm) to compare energy flow within the food webs. Model comparisons showed that the availability of food resources was the main factor influencing the biomass of Indo-Pacific humpback dolphins. Specifically, its more frequent occurrence in Ex can be attributed to greater phytoplankton production and greater biomasses of macroinvertebrates and prey fish than in the other two areas. An increase in fishing activity might decrease the food availability and, consequently, the biomass of the dolphins. Although the decline in the dolphin population would increase the biomass of some prey fish species, local fishermen might not necessarily benefit from the decline due to the concurrent decrease of highly valued crabs and shrimp. Collectively, our work suggests that the Indo-Pacific humpback dolphin is a keystone species in tropical coastal waters of Taiwan, and thereby exhibit a disproportional large ecological impact given their relatively low abundance.

## Introduction

The Indo-Pacific humpback dolphin (*Sousa chinensis*), also known as the Chinese white dolphin, is mostly observed within 400 m of shore throughout the Indian and Western Pacific oceans [1]. The Indo-Pacific humpback dolphin is a strictly coastal species and prefers estuarine habitats for feeding [2–4]. As a top predator of coastal ecosystems, it can remain in shallow inshore waters to increase the availability of food sources [5]. The extent of the use/residence time of an estuarine habitat by the Indo-Pacific humpback dolphin is greatly influenced by the tide-driven activity of prey fish [6]; likewise, high volumes of river discharge can move its prey seaward from the estuary [7], which also affects the time these dolphins spend in the estuary themselves.

Indo-Pacific humpback dolphins are critically endangered and have been at the forefront of a number of conservation issues [8–11]. Although estuarine habitats are known to be their preferred feeding grounds, the trophic relationships between the Indo-Pacific humpback dolphin and other organisms within coastal ecosystems remain virtually unknown. Efforts to conserve a given species are often hindered by a limited understanding of the dynamics of the trophic structure and energy flow network within which said species lives [12–13]. As such, an understanding of these trophic relationships could potentially increase our ability to explain the abundance and distribution of the Indo-Pacific humpback dolphin, as well as to predict its population dynamics under periods of future environmental change. Trophic models have been constructed to explore the possible reasons for the population dynamics of Steller sea lions in Alaska [14]. In addition to environmental variation, killer whale predation, and competition with Pacific halibut, their simulations indicated that overfishing of mackerel and herring was an important factor in determining the sea lion population dynamics. Similarly, a trophic model of a Galápagos rocky reef system comprising the Galápagos sea lion was constructed to explore trophic relationships within the system and potential solutions to overfishing [15].

In Taiwan, direct fishing for dolphins is prohibited. However, because feeding habit studies found that Indo-Pacific humpback dolphins and fishermen can target the same species [16], competition may exist between fishermen and Indo-Pacific humpback dolphins for the same fisheries resources. There is recent evidence suggesting that the fisheries resources in the coastal waters of Taiwan have declined [17]. This would detrimentally affect not only the fishermen but also Indo-Pacific humpback dolphins. There is therefore a need to evaluate the risk of this potential threat and establish a level of fishing activity that allows for the conservation of the Indo-Pacific humpback dolphin.

The Xin-Huwei River Estuary (Ex) of Western Taiwan has been identified as a “hotspot” for sightings of Indo-Pacific humpback dolphins, though the nearby Zhuoshui River Estuary (Ez) is not [7]. To explore the reasons underlying the abundance of Indo-Pacific humpback dolphins in Ex and to evaluate its trophic significance, we constructed trophic models of these two tropical estuaries (Ex and Ez) and compared them to an offshore site (Dm) in Western Taiwan. We hypothesized that the higher phytoplankton production in Ex [18] may have led to a higher abundance of prey species of Indo-Pacific humpback dolphins, and consequently higher abundance of the dolphins themselves.

## Materials and Methods

### Study area

**Fig 1:**
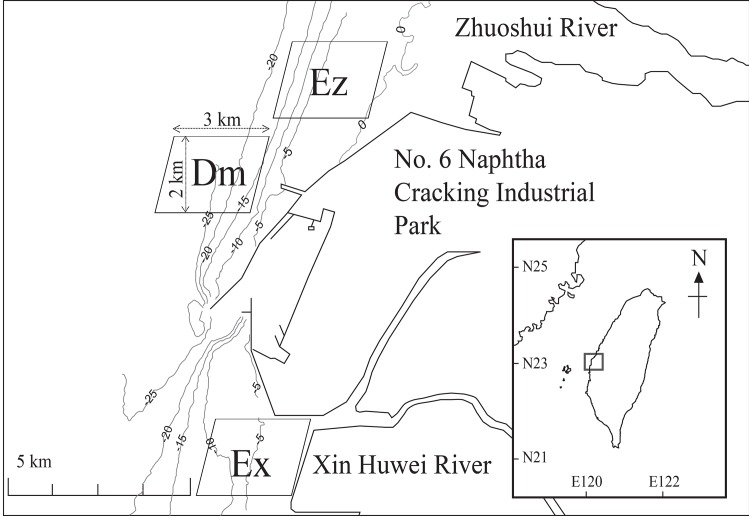
Study areas. Study areas are shown in parallelograms in the coastal waters of Western Taiwan, with a map of Taiwan provided in the inset. No. 6 Naphtha Cracking Industrial Park was constructed in 1994 and launched commercially operations in 1998.

Ez and Ex are located off the west coast of central Taiwan (Fig 1), where the shallow-sloped coastline is typically characterized by muddy flats. The average water temperature over the year is 24.1°C, with an average annual rainfall of 2,384 mm (1998–2014, the Central Weather Bureau of Taiwan; http://www.cwb.gov.tw/V7/climate/monthlyMean/Taiwan_tx.htm). The wet season is from April to September, and the dry season lasts from October to March. According to the water quality data of the Environmental Protection Agency (EPA) of Taiwan (2012–2013) and our own prior research [18], the mean suspended solid concentration and turbidity in Ez reached 894.7 mg L^-1^ and 11.7 NTU due to the high content of slate clay in the river. The mean concentrations of NH_4_, NO_x_ (NO_2_+NO_3_), and total phosphorus (TP) in Ez were 4.29, 12.89, and 5.24 μM, respectively. The mean suspended solid concentration and turbidity was much lower (168.2 mg L^-1^ and 7.2 NTU) in Ex, but the mean nutrient concentrations were higher (the concentrations of NH_4_, NO_x_, and TP were 219.6, 96.5, and 7.81 μM, respectively) because of the deposition of piggery sewage in the catchment of Ex. The offshore site Dm was relatively less influenced by the catchment runoff [18].

We constructed trophic models of the three study areas in coastal Western Taiwan (Fig 1). Each area was a parallelogram with a height of 2 km and a width (base) of 3 km. Ez and Ex were located at approximately 2 km offshore of the Zhuoshui River Estuary (23°50'49" N, 120°11'18" E) and the Xin Huwei River Estuary (23°44'54" N, 120°9'38" E), respectively. The water depth of the two areas was 7–8 m. The offshore site (Dm; 23°49'11" N, 120°9'28" E) was located between the two estuaries; specifically, it was 3 km outside of the No. 6 Naphtha Cracking Industrial Park and had an average depth of 21 m.

### Modeling approach

Trophic models of the three study areas were constructed using the Ecopath routine in the Ecopath with Ecosim software system (version 6.2 [19]) to quantify energy flow in the food web. For each compartment *i*, a mass-balance budget was expressed as:

Pi−BiM2i−Pi×(1−EEi)−EXi−ACi=0 (1)

where P is production, B is biomass, M2 is predation mortality, EE is ecotrophic efficiency (i.e., the portion of the production that is either passed up to the next higher trophic level or exported), 1 − EE is “other mortality”, EX is the export to other systems, and AC is biomass accumulation during the study period.

A predator group *j* is connected to its prey group *i* by its consumption. Thus, Eq (1) can be re-expressed as:

Bi×Pi/Bi×EEi−ΣjBj×Qj/Bj×DCji−EXi−ACi=0 (2)

where P*i*/B*i* is the production/biomass ratio of prey *i*, Q*j*/B*j* is the consumption/biomass ratio of predator *j*, and DC*ji* is the fraction of *i* in the average diet of *j*. It was assumed that the food matrix remained stable during the study period (2012–2013). Because biomass and/or abundance of phytoplankton, zooplankton, invertebrates, fish, and the Indo-Pacific humpback dolphin appeared to be unchanged during the study period (data not shown), it was also assumed that EX and AC for the major compartments were zero.

Consumption of *j* is then connected to its production, which can be re-expressed as:

ΣjBj×Qj/Bj=Pj+Rj+UNj (3)

where R is respiration and UN is unused consumption, which was assumed to be 20% [19].

### Model compartments

Major species of similar sizes and diets in the study area were functionally grouped within the same compartment. A 19-compartment model was developed for each study area: (1) Indo-Pacific humpback dolphins (*S*. *chinensis*), (2) pelagic piscivorous fish (mainly *Terapon jarbua* and *Trichiurus lepturus*), (3) benthic piscivorous fish (mainly *Dasyatis akajei* and *D*. *bennettii*), (4) large benthic-feeding fish (mainly *Arius* sp.), (5) small benthic-feeding fish (mainly *Cynoglossus bilineatus* and *Paraplagusia blochii*), (6) zooplanktivorous fish (mainly *Pelates quadrilineatus* and *Ilisha elongata*), (7) omnivorous fish (mainly *Acanthopagrus berda* and *A*. *schlegelii*), (8) cephalopods (mainly *Loliolus* sp.), (9) stomatopods (mainly *Oratosquillina interrupta*), (10) crabs (mainly *Portunus pelagicus*), (11) shrimp (mainly *Parapenaeopsis hardwickii*), (12) gastropods (mainly *Hemifusus tuba*), (13) bivalves (mainly *Corbula fortisulcata*), (14) amphipods, (15) polychaetes (mainly members of the Terebellidae family), (16) carnivorous zooplankton (mainly chaetognathids), (17) herbivorous zooplankton (mainly members of the *Noctiluca* and *Calanus* genera), (18) phytoplankton (mainly diatoms), and (19) organic detritus. Bacterial processes are difficult to estimate reliably, and their energy flows might completely overshadow others within the system [19]. It was assumed that bacteria were associated with organic detritus; therefore, they are linked to the present model only through detrital import and export by bacterial production and respiration. As only a few seabirds were observed flying above the sea surface during the study period, the export of fish and invertebrates by birds was assumed to be small when compared with those by fisheries and was not included in the models.

### Sampling and parameterization

Main parameters (e.g., biomass, primary production, and diet composition) used to construct the Ecopath models were assembled from our own studies covering all three study areas and two cycles of seasonality from winter 2012 to fall 2013 (Tables 1–3 and S1–S5 Tables). All sampling procedures were specifically approved by the Coast Guard Administration of Taiwan in accordance with the pre-approved permit. According to the long-term record of water temperature in the study areas (1998–2014, the Central Weather Bureau of Taiwan; http://www.cwb.gov.tw/V7/climate/marine_stat/wtmp.htm), we conducted sampling during December and March (<20°C) for winter, April and May (from 20°C to 28°C) for spring, June and September (>28°C) for summer, and October and November (from 28°C to 20°C) for fall. Spring and fall in Taiwan span only 1–2 months.

**Table 1:** Compartments, input parameters, and estimated output parameters (bold) for the Ecopath model in Ez.

	Group name	B	P/B	Q/B	Y	TL	EE	P/Q	NE	R
1	Indo-Pacific humpback dolphins	0.0045	0.11	12.9	--	**3.77**	**0.00**	**0.01**	**0.01**	**0.05**
2	Pelagic piscivorous fish	0.0202	0.53	7.10	0.008	**2.92**	**0.97**	**0.07**	**0.09**	**0.10**
3	Benthic piscivorous fish	0.0954	0.47	4.51	0.001	**3.04**	**0.99**	**0.10**	**0.13**	**0.30**
4	Large benthic-feeding fish	0.1485	0.71	5.59	0.050	**2.81**	**0.98**	**0.13**	**0.16**	**0.56**
5	Small benthic-feeding fish	0.0911	0.85	20.9	0.020	**2.44**	**0.99**	**0.04**	**0.05**	**1.45**
6	Zooplanktivorous fish	0.0061	0.89	7.03	0.002	**2.78**	**0.97**	**0.13**	**0.16**	**0.03**
7	Omnivorous fish	0.0002	2.77	61.5	--	**2.51**	**0.99**	**0.05**	**0.06**	**0.01**
8	Cephalopods	0.0010	2.41	16.6	0.001	**2.71**	**0.97**	**0.15**	**0.18**	**0.01**
9	Stomatopods	0.0001	3.54	14.2	--	**2.36**	**0.93**	**0.25**	**0.31**	**0.00**
10	Crabs	0.0085	2.96	11.6	0.001	**2.19**	**0.97**	**0.25**	**0.32**	**0.05**
11	Shrimp	0.0651	3.56	19.0	--	**2.26**	**0.91**	**0.19**	**0.23**	**0.76**
12	Gastropods	0.0264	2.33	7.70	--	**2.16**	**0.97**	**0.30**	**0.38**	**0.10**
13	Bivalves	0.0886	2.78	9.50	--	**2.00**	**0.99**	**0.29**	**0.37**	**0.43**
14	Amphipods	0.0004	14.0	33.4	--	**2.04**	**0.50**	**0.42**	**0.53**	**0.00**
15	Polychaetes	0.2865	5.53	24.2	--	**2.01**	**0.62**	**0.23**	**0.29**	**3.96**
16	Carnivorous zooplankton	0.0212	6.35	30.0	--	**2.90**	**0.83**	**0.21**	**0.26**	**0.37**
17	Herbivorous zooplankton	0.0903	42.1	95.0	--	**2.00**	**0.31**	**0.44**	**0.55**	**3.06**
18	Phytoplankton	1.6509	1.00	--	--	**1.00**	**0.95**	**--**	**--**	**--**
19	Detritus	329.40	--	--	--	**1.00**	**0.98**	**--**	**--**	**--**

B: biomass (g WW m^-2^); P/B: production/biomass (yr^-1^); Q/B: consumption/biomass (yr^-1^); Y: fishery catch rate (g WW m^-2^ yr^-1^); TL: trophic level; EE: ecotrophic efficiency; NE: net efficiency (P/(P + R)); R: respiration (g WW m^-2^ yr^-1^).

**Table 2:** Compartments, input parameters, and estimated output parameters (bold) for the Ecopath model in Dm.

	Group name	B	P/B	Q/B	Y	TL	EE	P/Q	NE	R
1	Indo-Pacific humpback dolphins	0.0060	0.11	12.9	--	**3.69**	**0.00**	**0.01**	**0.01**	**0.06**
2	Pelagic piscivorous fish	0.0044	0.53	7.10	0.001	**3.05**	**0.94**	**0.07**	**0.09**	**0.02**
3	Benthic piscivorous fish	0.0443	0.47	4.51	0.001	**3.03**	**0.92**	**0.10**	**0.13**	**0.14**
4	Large benthic-feeding fish	0.1192	0.71	5.59	0.030	**2.83**	**0.93**	**0.13**	**0.16**	**0.45**
5	Small benthic-feeding fish	0.1080	0.85	20.9	0.020	**2.44**	**0.98**	**0.04**	**0.05**	**1.72**
6	Zooplanktivorous fish	0.0010	0.89	7.03	<0.001	**2.78**	**0.95**	**0.13**	**0.16**	**<0.01**
7	Omnivorous fish	<0.0001	2.77	61.5	<0.001	**2.51**	**0.93**	**0.05**	**0.06**	**<0.01**
8	Cephalopods	0.0008	2.41	16.6	0.001	**2.71**	**0.93**	**0.15**	**0.18**	**0.01**
9	Stomatopods	0.0026	3.54	14.2	--	**2.36**	**0.93**	**0.25**	**0.31**	**0.02**
10	Crabs	0.0181	2.96	11.6	0.001	**2.19**	**0.95**	**0.25**	**0.32**	**0.11**
11	Shrimp	0.0489	3.56	19.0	--	**2.26**	**1.00**	**0.19**	**0.23**	**0.57**
12	Gastropods	0.0264	2.33	7.70	--	**2.16**	**0.92**	**0.30**	**0.38**	**0.10**
13	Bivalves	0.0886	2.78	9.50	--	**2.00**	**0.81**	**0.29**	**0.37**	**0.43**
14	Amphipods	0.0001	14.0	33.4	--	**2.04**	**0.95**	**0.42**	**0.53**	**<0.01**
15	Polychaetes	0.1922	5.53	24.2	--	**2.01**	**0.95**	**0.23**	**0.29**	**2.66**
16	Carnivorous zooplankton	0.4236	6.35	30.0	--	**2.90**	**0.03**	**0.21**	**0.26**	**7.48**
17	Herbivorous zooplankton	0.6307	42.1	95.0	--	**2.00**	**0.44**	**0.44**	**0.55**	**21.36**
18	Phytoplankton	1.3042	1.00	--	--	**1.00**	**0.92**	**--**	**--**	**--**
19	Detritus	174.70	--	--	--	**1.00**	**0.98**	**--**	**--**	**--**

Please see Table 1 for the full names of the parameters in the top-most row.

**Table 3:** Compartments, input parameters, and estimated output parameters (bold) for the Ecopath model in Ex.

	Group name	B	P/B	Q/B	Y	TL	EE	P/Q	NE	R
1	Indo-Pacific humpback dolphins	0.0200	0.11	12.9	--	**3.82**	**0.00**	**0.01**	**0.01**	**0.20**
2	Pelagic piscivorous fish	0.0411	0.52	6.84	0.008	**3.05**	**0.99**	**0.08**	**0.10**	**0.20**
3	Benthic piscivorous fish	0.1185	0.59	5.44	0.001	**3.08**	**0.93**	**0.11**	**0.14**	**0.44**
4	Large benthic-feeding fish	0.3024	0.86	9.54	0.050	**2.89**	**0.99**	**0.09**	**0.11**	**2.05**
5	Small benthic-feeding fish	0.1574	0.87	19.4	0.020	**2.44**	**0.99**	**0.04**	**0.06**	**2.30**
6	Zooplanktivorous fish	0.0125	0.64	7.69	0.002	**2.78**	**0.97**	**0.08**	**0.10**	**0.07**
7	Omnivorous fish	0.0120	0.45	4.83	0.001	**2.51**	**0.98**	**0.09**	**0.12**	**0.04**
8	Cephalopods	0.0040	2.44	16.6	0.001	**2.98**	**0.97**	**0.15**	**0.18**	**0.04**
9	Stomatopods	0.0020	3.50	13.9	--	**2.37**	**0.91**	**0.25**	**0.31**	**0.02**
10	Crabs	0.0752	3.04	11.6	0.001	**2.19**	**0.95**	**0.26**	**0.33**	**0.47**
11	Shrimp	0.1386	3.61	19.0	--	**2.27**	**0.99**	**0.19**	**0.24**	**1.61**
12	Gastropods	0.0380	2.26	7.70	--	**2.14**	**0.94**	**0.29**	**0.37**	**0.15**
13	Bivalves	0.3955	1.99	9.50	--	**2.00**	**0.98**	**0.21**	**0.26**	**2.22**
14	Amphipods	0.0005	13.9	33.4	--	**2.04**	**0.85**	**0.42**	**0.52**	**0.01**
15	Polychaetes	0.2862	5.49	24.2	--	**2.01**	**1.00**	**0.23**	**0.28**	**3.97**
16	Carnivorous zooplankton	0.1605	6.35	30.0	--	**2.90**	**0.79**	**0.21**	**0.26**	**2.83**
17	Herbivorous zooplankton	0.2466	40.4	95.0	--	**2.00**	**0.53**	**0.43**	**0.53**	**8.77**
18	Phytoplankton	1.5830	74.7	--	--	**1.00**	**0.12**	**--**	**--**	**--**
19	Detritus	392.40	--	--	--	**1.00**	**0.22**	**--**	**--**	**--**

Please see Table 1 for the full names of the parameters in the top-most row.

Indo-Pacific humpback dolphin abundance was estimated via line transect surveys on fishing boats. In total, we conducted 48 surveys across the three study areas during the study period (12, 28, 4, and 4 in spring, summer, autumn, and winter, respectively). The boat had an open upper deck to allow observers’ line of sight to be positioned approximately 4–5 m above sea level. To keep track of individual dolphins, we identify them by their unique dorsal fin characteristics, such as shape and notches. On each survey, the boat traveled along pre-determined transects averaging 69.14±7.53(std. dev.) km at a speed of approximately 8 km h^-1^. The abundance was estimated using the program DISTANCE 5.0 [20], and the abundance data were converted to biomass per unit area by multiplying by the average adult biomass of 180 kg [21] and dividing by its home range of 200 km^2^ [4]. The P/B and Q/B values of the Indo-Pacific humpback dolphin were derived from empirical relationships from prior works ([22] and [23], respectively). The diet composition of the Indo-Pacific humpback dolphin was assumed to be similar to those of others assessed previously in Western Taiwan [24], as determined by stomach content analysis.

Eight sampling/survey events were performed to estimate the seasonal abundance of fish, macroinvertebrate, zooplankton, and phytoplankton in the three study areas during the study period. Fish and macroinvertebrate samples were collected by a local commercial shrimp trawler with a mesh size of 3.5 cm in the cod end. The samples were first sorted into fish, shrimp, crabs, cephalopods, clams, snails, and others onboard and were further identified to the lowest taxon possible upon return to the laboratory. Infauna data were derived from the monitoring surveys performed in the same region by Taiwan’s EPA from 2012 to 2013 (http://www.epa.gov.tw/np.asp?ctNode=32999&mp=epa) using a grab sampling device (0.1 m^2^). P/B and Q/B values of fish were derived from FishBase (www.fishbase.org). The diet composition of the various fish species were assumed to be similar to those of conspecifics (or those of the same functional group when conspecific data were lacking) of the nearby Pearl River Estuary [25]. P/B values of the sampled invertebrates were estimated from the empirical equations of a prior work [26]. Q/B values and the diet composition of invertebrates were assumed to be similar to those of the same functional groups assessed in neighboring lagoons of Taiwan [27–28]. Fishery data from the three study areas were also derived from Taiwan’s EPA’s 2012–2013 monitoring surveys (http://www.epa.gov.tw/np.asp?ctNode=32999&mp=epa).

Zooplankton samples were collected by a NorPac net (45 cm in diameter with a 330-mm mesh size) positioned just below the sea surface for 5 min at a speed of 1.0 m s^-1^ during the same period at which shrimp trawling was performed (described above). All zooplankton samples were fixed with formalin onboard the boat. In the laboratory, the zooplankton samples were sorted into 34 taxonomic groups and quantified. Gelatinous zooplankton were picked up carefully to measure the settling volume and wet weight, whereas other zooplankton was dried in an oven at 60°C for 48 h for dry weight measurements. Q/B values were obtained from the literature on tropical coastal systems [27, 29]. P/B values of carnivorous and herbivorous zooplankton were derived from the empirical equations of Hirst et al. [30] and Hirst and Bunker [31], respectively. The dietary compositions of zooplankton were assumed to be similar to those assessed in zooplankton sampled from a nearby lagoon [28]. There are few published works on the diets of small benthic invertebrates, so this information was obtained by searching for the same groups/species sampled and analyzed from tropical waters elsewhere in the world [32].

The phytoplankton biomass and productivity data were derived from our own prior research in the study areas [18]. Phytoplankton biomass, in terms of chlorophyll *a* (CHL *a*) concentration (corrected for phaeopigment), was determined on a Turner fluorometer (Trilogy, Turner Design, USA). Phytoplankton productivity was determined using a dissolved oxygen (DO)-based method by incubating 300-mL BOD bottles in outdoor flowing seawater tanks. This incubation was conducted *in situ* on the coast next to Ez on a sunny day under ambient light from 10:00 h to 14:00 h, when irradiance (1500–2000 μmol m^–2^ s^–1^) was at saturating levels for photosynthesis. The BOD bottles were exposed in the field to five different irradiances of 0, 30, 50, 70, or 100% shading by interposing screens with different mesh sizes (n = 3 for each irradiance per study area). The net production (NP) rates under various irradiances and respiration rates (100% shading or opaque bottles) were derived from changes in the DO concentrations over time measured by a DO meter (Model 52, 5909 probe, YSI, USA). The gross production (GP) rate was then calculated as the sum of the respiration and NP rates. A ratio of 1.48 between respiration rates during daytime and nighttime periods [33] was used to calibrate nighttime respiration rates.

Daily phytoplankton GP rates were calculated by integrating the interpolated GP rates under various irradiances for each seasonal incubation in each study area using the surface light data at the time of measurement. This analysis also considered the relationship between irradiance and GP (P-E curve). For each seasonal incubation, incident photosynthetically active radiation (PAR) was measured *in situ* at 5-min intervals with a Li-1400 meter (LI-COR, USA) from 07:00 h until after sunset (19:00 h). For each study area, annual phytoplankton NP rates were then calculated via integration of daily GP and respiration rates in different seasons by considering the duration of each season. Rates of oxygen production were converted to carbon fixation rates using a photosynthetic quotient of 1.2 [34].

Detritus samples were collected in the three study areas seasonally from January to August 2014. Surface water was filtered through acid-cleaned, dried, and pre-weighed 0.7 μm GF-75 filters (AdvanTec, Japan) and then incinerated in a muffle furnace at 450°C for 4 h to determine the organic detrital mass.

Factors used to interconvert between CHL *a*, carbon, displacement volume, dry weight, and wet weight were based on the table summarized by Opitz [32]. Biomass data were then recorded as grams of wet weight (WW) per square meter (g WW m^-2^), and flow data were recorded as grams of wet weight per square meter per year (g WW m^-2^ yr^-1^).

### Model balancing and verification

Because Ecopath establishes links between the production of one compartment and the consumption of other compartments to calculate one missing parameter for each group, the least certain parameter from each compartment can be treated as unknown and calculated by Ecopath [19]. In this study, the biomass of each compartment and primary production were considered the most reliable data. The EE was treated as unknown and left for Ecopath to estimate in order to verify the realism of the models. Some P/B, Q/B, and DC values of fish and invertebrates were assembled from the literature and were also considered to be less reliable. Therefore, their values were gradually modified during the model mass-balancing exercise. However, most of the changes were rather small and remained within 20% of the input value (S1 Table). The pedigree routine was used to determine an overall index of model quality [35] based on the origin and quality of each model input parameter. The pedigree index varies from 1.0 for a high-quality model whose inputs are based on locally, well-sampled, high-precision data to 0.0 when parameters are estimated or taken from other models described in the literature.

### Network analysis

Transfer details of organic matter from primary producers and organic detritus to top predators within the food web can be revealed using network analysis [36]. Mixed trophic impacts [37] were calculated to assess the direct and indirect impacts of a change in the biomass of each compartment on the other compartments. Keystone species are defined as relatively low biomass species with a high impact on other components within the food web [38], and the “keystoneness” of each compartment was estimated by applying the results of mixed trophic impacts [39]. The sum of consumption, exports, fisheries catch, respiratory flows, and flows into detritus was indexed as the total system throughput, indicating how much organic matter a system processed. The Finn cycling index [40] was used to measure how retentive the system was, and Lindeman trophic analysis [40] was applied to simplify the complex food web into a single, linear food chain. The trophic efficiency of the transfer from one aggregated trophic level to the next was calculated as the fraction of the input of organic matter to a given level that was transferred to the next higher level.

## Results

### Model balancing and verification

A four-category scale of pedigree index was proposed by Morissette [41]: <0.2, 0.2–0.399, 0.4–0.599, ≥0.6; the last category being termed “very high pedigree”. The pedigree index was 0.67 for the models, indicating a sufficient quality to make ecosystem inferences.

**Fig 2:**
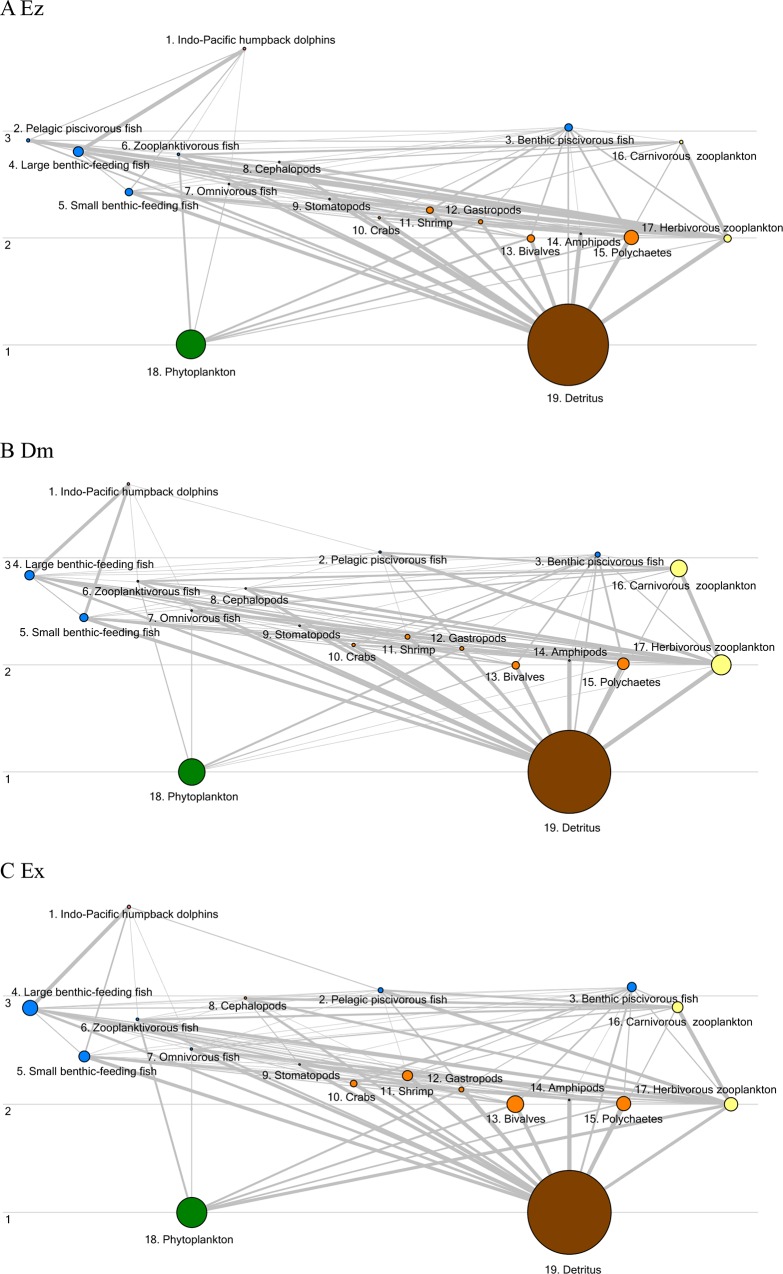
**Flow diagrams for the (A) Ez, (B) Dm, and (C) Ex models.** The circle sizes are proportional to the compartmental biomass, and the thicknesses of the lines are proportional to the energy flow rate.

The food webs in the three study areas rely on two major food sources: autochthonous phytoplankton production within the system and allochthonous organic detritus transferred from other systems (Fig 2). Phytoplankton NP rates were much greater in Ex than in Ez and Dm. The NP rates in Ez and Dm were negative due to the very high respiration rates [18], suggesting that both systems were heterotrophic. Therefore, more organic matter in the systems was consumed than was produced by phytoplankton. The P/B values of phytoplankton were assumed to be 1.0 in both areas, indicating that plankton production was approximately equal to the biomass required to maintain phytoplankton biomass during the study period (Tables 1–3). This assumption was validated by our own field studies [18], in which phytoplankton biomass did not change significantly during the study period. The additional organic matter needed to sustain the two systems was mainly derived from the massive subsidy of organic detritus from terrestrial sources in the catchments [18]. To balance the model, the input of organic detritus to the system from the catchment was increased to 10 g WW m^‐2^ yr^‐1^ and 35 g WW 274 m^‐2^ yr^‐1^ in Ez and Dm, respectively, to meet the need for the consumption of additional organic matter within the food web during the mass-balancing exercise.

In total, there were 36 individuals of the Indo-Pacific humpback dolphin observed in the study areas. The occurrence of the Indo-Pacific humpback dolphin derived from our transect surveys was 5, 7, and 24 individuals in Ez, Dm, and Ex, respectively. The abundance estimates were transformed to biomass per square meter based on the mean individual biomass and its home range (described above) for the model inputs (0.0045, 0.0060, and 0.020 g WW m^-2^ in Ez, Dm, and Ex, respectively)(Tables 1–3).

To verify the realism of the Ecopath models, the EE and GE (the gross food conversion efficiency, i.e., the P/Q) values were examined. The EE values of all compartments of the Ecopath models were < 1 (Tables 1–3), and the P/Q values of most of the compartments were between 0.05 and 0.30. The P/Q value of Indo-Pacific humpback dolphins was < 0.05, as they are the top predators of the systems. The growth rates of small-sized amphipods and herbivorous zooplankton were relatively high, so the P/Q values were > 0.30. In addition, the P/Q values of all the compartments were not larger than the NE values (the production divided by the assimilated portion of the food, i.e., P/(P + R)). These outputs indicate that the Ecopath models were realistic.

### Trophic structure and energy flow

The trophic structures of the Ecopath models of the three study areas were similar, but the compartmental biomasses differed (Fig 2). The effective trophic levels of Indo-Pacific humpback dolphins ranked the highest (the fourth integrated trophic level, [TL IV]; ranging from 3.69–3.82), followed by carnivorous zooplankton, cephalopods, and fish (TL III), and then by herbivorous zooplankton and most of the benthic invertebrates (TL II). The biomasses of carnivorous and herbivorous zooplankton were higher in Dm, whereas the biomasses of fish and macroinvertebrates were higher in Ex (Tables 1–3).

Comparisons of energy flow within the three trophic models further showed that total system throughput and NPP were much greater in Ex than in the other two areas (Table 4). The TPP/TR ratio was also much higher in Ex than in the other two areas. Consequently, net system production values were negative in the heterotrophic systems of Ez and Dm. Total biomass and total consumer biomass were also greater in Ex. The sum of all consumption and the sum of all respiratory flows were higher in Dm. Energy flow and total biomass were markedly lower in Ez than in the other two areas.

**Table 4:** Ecosystem statistics of the Ez, Dm, and Ex models.

Parameter	Ez	Dm	Ex	Unit
Sum of all consumption	21.98	82.76	50.10	g WW m^-2^ yr^-1^
Sum of all exports	0.40	1.17	92.9	g WW m^-2^ yr^-1^
Sum of all respiratory flows	11.25	35.14	25.39	g WW m^-2^ yr^-1^
Sum of all flows into detritus	17.78	69.17	119.5	g WW m^-2^ yr^-1^
Total system throughput	51.41	188.2	287.9	g WW m^-2^ yr^-1^
Sum of all production	7.99	32.3	133.0	g WW m^-2^ yr^-1^
Total biomass (excluding detritus)	2.60	3.02	3.59	g WW m^-2^
Total biomass of all consumers	0.95	1.72	2.01	g WW m^-2^
Calculated total net primary production (NPP)	1.65	1.30	118	g WW m^-2^ yr^-1^
Total primary production/total respiration (TPP/TR)	0.15	0.04	4.66	
Net system production	-9.60	-33.8	92.9	g WW m^-2^ yr^-1^

### Network analysis

**Fig 3:**
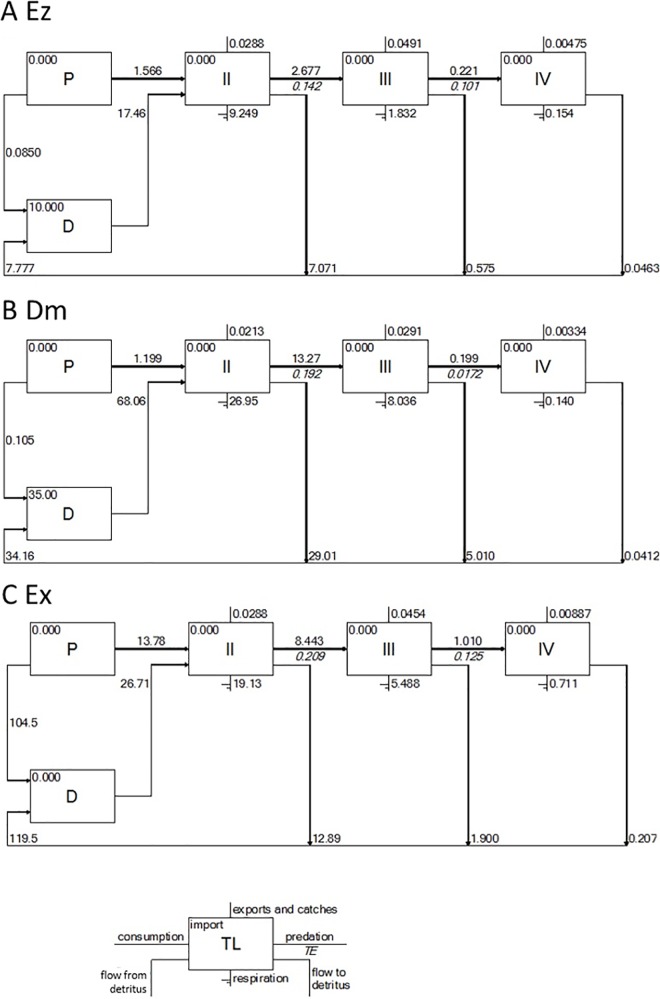
**Lindeman spines for the (A) Ez, (B) Dm and (C) Ex models.** The compartments were aggregated into trophic levels II-IV, and energy flows were derived from primary producers (P) or organic detritus (D).

The Lindeman spine of the three trophic models showed higher proportions of energy flows from organic detritus than from primary production at TL II, indicating the relative importance of detritus as a food source in the study areas, especially in the heterotrophic systems of Ez and Dm (Fig 3A and 3B). In Ex (Fig 3C), however, the proportions of energy flows from primary production and detritus were more similar to each other (approximately 2-fold difference in Ex rather than approximately 10- and 55-fold difference in Ez and Dm, respectively).

Because herbivorous and carnivorous zooplankton biomasses were greater in Dm, both total consumption and respiration were higher there. However, the energy did not flow up to TL IV (Fig 3B). Instead, energy flow into TL IV was 4-fold higher in Ex than in the two other areas (Fig 3C) because the feeding by Indo-Pacific humpback dolphins was more intense. Consequently, the trophic efficiencies were equivalent from TL II to III in all areas, but the trophic efficiency was much higher from the TL III to IV in Ex. The average trophic efficiency of the three trophic models was highest in Ex (13.4%), followed by Ez (10.9%) and Dm (6.6%).

### Mixed trophic impacts

**Fig 4:**
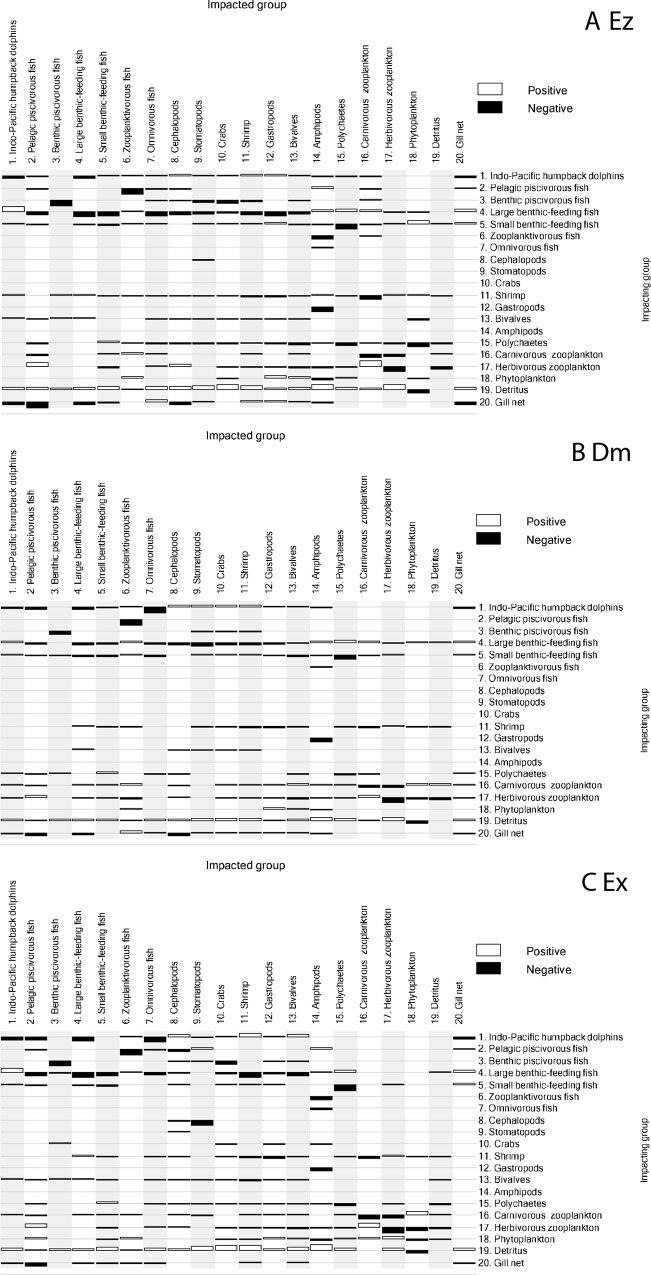
**Mixed trophic impacts of the (A) Ez, (B) Dm, and (C) Ex models**. Impacting groups were arranged in the right column, and impacted groups were represented as numbers above the column. Black and white bars indicate negative and positive impacts, respectively. No bar indicates little impact.

The mixed trophic impacts analysis revealed direct and indirect trophic interactions between Indo-Pacific humpback dolphins and other compartments in the three trophic models (Fig 4). Large benthic-feeding fish had the greatest positive impact on Indo-Pacific humpback dolphins because they were the dolphins’ main prey (S2–S4 Tables). Small benthic-feeding fish were also prey for these dolphins, but their contribution to the dolphins’ dietary composition was lower. Organic detritus was the main energy source of benthic food chains so an increase in the amount would indirectly increase the biomass of Indo-Pacific humpback dolphins. Indo-Pacific humpback dolphins had a negative impact on themselves, indicating that their biomass might be constrained by limited food resources in the study areas. Fisheries (shown as Gill net) had an intermediate negative impact on Indo-Pacific humpback dolphins in the three study areas.

The trophic significance of Indo-Pacific humpback dolphins is revealed by the mixed trophic impacts analysis (Fig 4). In the three study areas, Indo-Pacific humpback dolphins had direct negative impacts on their prey: pelagic piscivorous fish, large benthic-feeding fish, and omnivorous fish. Indo-Pacific humpback dolphins also had negative impacts on their competitors, including themselves and the fisheries. They had positive impacts on small benthic-feeding fish, cephalopods, stomatopods, crabs, shrimp, gastropods, and bivalves because the dolphins preyed upon the predators of these taxa. The results of mixed trophic impacts showed the impacts by Indo-Pacific humpback dolphins on other compartments were broad but were not negative for all groups.

**Fig 5:**
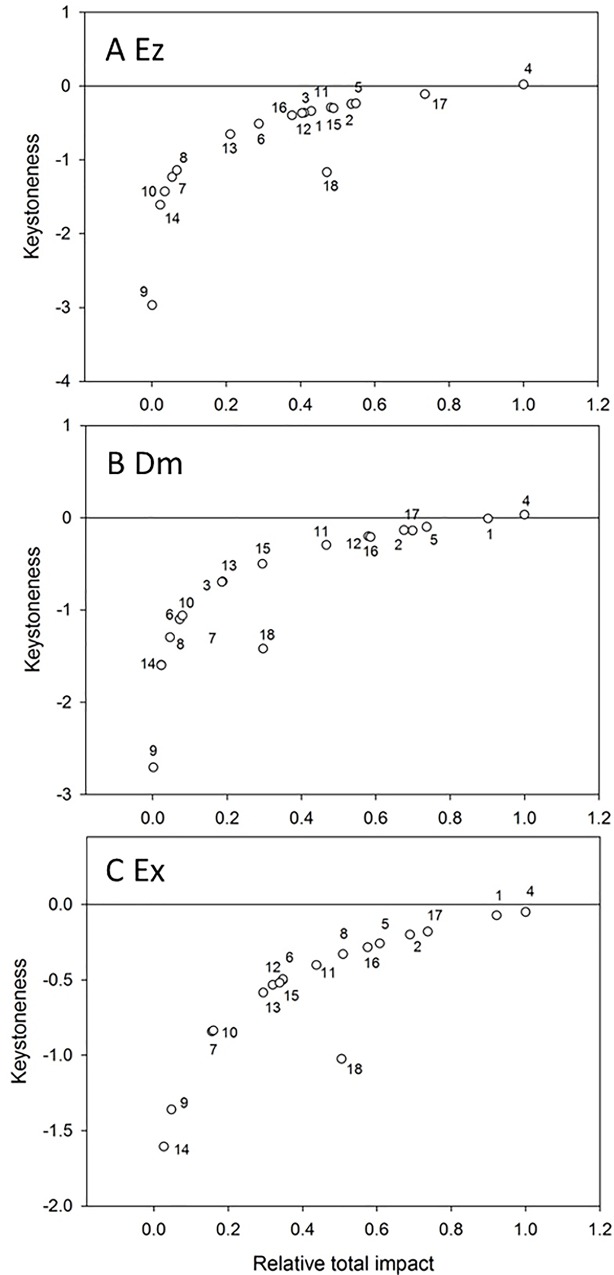
**Keystone index for each compartment in the (A) Ez, (B) Dm, and (C) Ex models**. 1: Indo-Pacific humpback dolphins; 2: Pelagic piscivorous fish; 3: Benthic piscivorous fish; 4: Large benthic-feeding fish; 5: Small benthic-feeding fish; 6: Zooplanktivorous fish; 7: Omnivorous fish; 8: Cephalopods; 9: Stomatopods; 10: Crabs; 11: Shrimp; 12: Gastropods; 13: Bivalves; 14: Amphipods; 15: Polychaetes; 16: Carnivorous zooplankton; 17: Herbivorous zooplankton; 18: Phytoplankton.

When the keystoneness of the functional groups is close to or larger than zero, they were the keystone groups within the food web. The keystoneness of Indo-Pacific humpback dolphins ranked intermediate in Ez, whereas the ranking in Dm and Ex increased to second amongst the functional groups (Fig 5), next to large benthic-feeding fish.

## Discussion

The trophic model of Ex revealed that the relatively high phytoplankton production resulted in greater abundance of macroinvertebrates and fish, especially large and small benthic-feeding fish, which were the main prey sources for Indo-Pacific humpback dolphins [16]. As Indo-Pacific humpback dolphins are often observed to alter their distribution during feeding/predation [5–7], the relatively higher phytoplankton production and more abundant prey species are likely the reason why the occurrence of Indo-Pacific humpback dolphins was higher in Ex. On the contrary, relatively lower phytoplankton production was associated with lower abundance of macroinvertebrates, fish, and Indo-Pacific humpback dolphins in Ez and Dm.

The heterotrophic systems of Ez and Dm were similar to Chesapeake Bay [42] and the Danshuei River Estuary [43], where a massive subsidy of organic detritus was also derived from terrestrial sources in the catchments. Although organic detritus can be a food source for many estuarine organisms, the quality of organic detritus may influence the system metabolism [44]. This is because organic detritus in estuaries is primarily derived from terrestrial plants of the catchment [45–46]. The organic matter therefore consists mainly of cellulose, and the carbon:nitrogen (C:N) ratio can exceed 150 [47], which is much higher than the Redfield C:N ratio (6.6) of phytoplankton [48]; this indicates that the nutrient content of organic detritus might be much less than that of phytoplankton production.

Turbidity has been identified as a main factor governing the seasonal and spatial variation in phytoplankton biomass and production in estuaries, in addition to nutrient concentrations [49]. The suspended solids and resuspended sediments in the river flows of estuaries can absorb and/or disperse irradiance in the water column and constrain phytoplankton production [50]. In estuaries with turbid water and high nutrient concentrations, the peaks of phytoplankton biomass and production often occur when irradiance is higher [51]. Our own prior research showed that there were highly negative correlations between turbidity and CHL *a* concentrations and the daily GP and NP rate in the study areas [18]. They further indicated the threshold of turbidity being 12 NTU, above which phytoplankton biomass and production rate were greatly reduced. The higher turbidity in Ez averaging 11.7 NTU might result in lower levels of phytoplankton biomass and production than in the other two study areas, despite high nutrient levels in the former.

Studies featuring trophic modeling of Indo-Pacific humpback dolphins are scarce, with the exception of one [25] performed in the Pearl River Estuary in 1981 and 1998 (Table 5). The TL and biomass of Indo-Pacific humpback dolphins were higher in the Pearl River Estuary in 1981 [25] and in Ex in this study (Table 5). Although the NPP of the Pearl River Estuary in 1981 was 14 times the level in Ex in 2012–2013, the fisheries catch in the Pearl River Estuary in 1981 was 20-fold higher than Ex. In addition to Indo-Pacific humpback dolphins, sharks and lizardfish were also the top predators in the Pearl River Estuary nearly thirty years ago. The comparable TL and biomass levels of Indo-Pacific humpback dolphins between the Pearl River Estuary in 1981 [25] and Ex in this study were possibly due to the intense competition with fishermen and marine competitors (i.e., sharks and lizardfish) in the Pearl River Estuary in 1981. In 1998, fishing activity and fisheries catch increased remarkably, and the total biomass of the system decreased 46% [25]. This may be the reason for the decline in dolphin biomass in the Pearl River Estuary in 1998, which is similar to the biomass in Ez and Dm herein, compared to 1981. On the other hand, the decrease in NPP of 45% in 1998 was possibly one of the reasons for the lower biomass of prey fish and Indo-Pacific humpback dolphins. Overall, the model comparisons showed that availability of food resources were likely the main factor influencing the biomass of Indo-Pacific humpback dolphins because the 1) decrease in NPP or 2) increase in fishing activity and/or competitors might decrease the food for Indo-Pacific humpback dolphins.

**Table 5:** Comparisons of trophic level (TLc) and biomass (Bc; g WW m^-2^) data for the Indo-Pacific humpback dolphin, as well as net primary production (NPP; g WW m^-2^ yr^-1^) and fisheries yield (Y; g WW m^-2^ yr^-1^), in the Pearl River Estuary and our study areas.

Site	TL_c_	B_c_	NPP	Y	Reference
Pearl River Estuary-1981	4.06	0.0195	1681	1.57	[25]
Pearl River Estuary-1998	3.59	0.0070	749	3.49	[25]
Ez	3.77	0.0045	1.65	0.08	this study
Dm	3.69	0.0060	1.30	0.05	this study
Ex	3.82	0.0200	118	0.08	this study

The mixed trophic impacts analysis also showed a close relationship between the biomass of Indo-Pacific humpback dolphins and fisheries. Although the results showed that a decrease in Indo-Pacific humpback dolphin abundance will increase the biomass of large benthic-feeding fish, pelagic piscivorous fish, and omnivorous fish, fishermen might not necessarily benefit from a decline in numbers of Indo-Pacific humpback dolphins. This is because the low economic value catfish *Arius* sp. was the large benthic-feeding fish that contributed most to the biomass of the group (62%). Catfish can secrete venom from a gland at the base of each pectoral spine, which may confer a human health risk [52]. In addition, the decline of Indo-Pacific humpback dolphins would lead to decreased biomasses of other fish and macroinvertebrates, especially crabs and shrimp. The most common crabs and shrimp were the Pacific blue swimming crab *Portunus pelagicus* (55% of the total abundance) and the sword prawn *Parapenaeopsis hardwickii* (44% of the total abundance), respectively, both of which are high value. It is clear that Indo-Pacific humpback dolphins had a substantial impact on abundance of other species, and therefore local fisheries, despite their relatively low abundance and biomass.

## Conclusions

The Ecopath models suggested that the higher occurrence of Indo-Pacific humpback dolphins in Ex was due to the higher phytoplankton production and greater prey abundance (e.g., macroinvertebrates and fish) than the other two study areas. We further found that the biomass of Indo-Pacific humpback dolphins was closely related to fisheries and competitors. Although a decrease in the numbers of Indo-Pacific humpback dolphins would increase the biomass of large benthic-feeding fish, pelagic piscivorous fish, and omnivorous fish, the decline would also lead to decreased biomasses of other fish and macroinvertebrates, especially high-economic-value crabs and shrimp. The fact that removal of even a small number of Indo-Pacific humpback dolphins results in such a large shift in the relative abundances of myriad other marine organisms supports its being labeled as a keystone species in the west coast of Taiwan.

## Supporting Information

S1 Table Original model input parameters before mass-balancing. (DOCX)

S2 Table Diet (proportional) composition matrix for the compartments in the Ez model. (DOCX)

S3 Table Diet (proportional) composition matrix for the compartments in the Dm model. (DOCX)

S4 Table Diet (proportional) composition matrix for the compartments in the Ex model. (DOCX)

S5 Table Source of model input parameters. (DOCX)
