# Supplementary material for: The Trophic Significance of the Indo-Pacific Humpback Dolphin, Sousa chinensis, in Western Taiwan
Source: PLoS One. 2016 Oct 25;11(10):e0165283. doi: 10.1371/journal.pone.0165283 (PMC5079652; doi:10.1371/journal.pone.0165283)
Supplement: S1 Table — (DOCX) [file pone.0165283.s001.docx]

**S1 Table.** **Original model input parameters before balancing.**

|  |  | B | | | P/B | | | Q/B | | |
| --- | --- | --- | --- | --- | --- | --- | --- | --- | --- | --- |
|  | Group name | Ez | Dm | Ex | Ez | Dm | Ex | Ez | Dm | Ex |
| 1 | Indo-Pacific humpback dolphins | 0.004 | 0.006 | 0.024 | 0.11 | 0.11 | 0.11 | 12.92 | 12.92 | 12.92 |
| 2 | Pelagic piscivorous fish | 0.020 | 0.004 | 0.041 | 0.53 | 0.53 | 0.52 | 7.10 | 7.10 | 6.84 |
| 3 | Benthic piscivorous fish | 0.095 | 0.044 | 0.119 | 0.46 | 0.46 | 0.59 | 4.51 | 4.51 | 5.44 |
| 4 | Large benthic-feeding fish | 0.149 | 0.119 | 0.302 | 0.71 | 0.71 | 0.86 | 5.59 | 5.59 | 9.54 |
| 5 | Small benthic-feeding fish | 0.091 | 0.108 | 0.157 | 0.85 | 0.85 | 0.87 | 20.97 | 20.97 | 19.36 |
| 6 | Zooplanktivorous fish | 0.003 | 0.001 | 0.010 | 0.88 | 0.89 | 0.64 | 7.03 | 7.03 | 7.69 |
| 7 | Omnivorous fish | 0.0002 | 0.0001 | 0.012 | 2.77 | 2.77 | 0.45 | 61.50 | 61.50 | 4.83 |
| 8 | Cephalopods | 0.001 | 0.001 | 0.004 | 2.41 | 2.41 | 2.44 | 16.60 | 16.60 | 16.60 |
| 9 | Stomatopods | 0.0001 | 0.003 | 0.002 | 3.54 | 3.54 | 3.50 | 17.70 | 17.70 | 17.50 |
| 10 | Crabs | 0.006 | 0.012 | 0.050 | 2.95 | 2.95 | 3.04 | 11.60 | 11.60 | 11.60 |
| 11 | Shrimp | 0.043 | 0.033 | 0.092 | 3.56 | 3.56 | 3.61 | 19.00 | 19.00 | 19.00 |
| 12 | Gastropods | 0.026 | 0.026 | 0.038 | 2.33 | 2.33 | 2.26 | 7.70 | 7.70 | 7.70 |
| 13 | Bivalves | 0.089 | 0.089 | 0.396 | 2.78 | 2.78 | 1.99 | 9.50 | 9.50 | 9.50 |
| 14 | Amphipods | 0.0004 | 0.0001 | 0.001 | 14.04 | 14.04 | 13.94 | 33.40 | 33.40 | 33.40 |
| 15 | Polychaetes | 0.286 | 0.192 | 0.286 | 5.53 | 5.53 | 5.49 | 24.20 | 24.20 | 24.20 |
| 16 | Carnivorous zooplankton | 0.021 | 0.424 | 0.161 | 6.35 | 6.35 | 6.35 | 30.00 | 30.00 | 30.00 |
| 17 | Herbivorous zooplankton | 0.090 | 0.631 | 0.247 | 42.12 | 42.12 | 40.43 | 95.00 | 95.00 | 95.00 |
| 18 | Phytoplankton | 1.406 | 1.304 | 1.583 | 1.00 | 1.00 | 74.74 | -- | -- | -- |
| 19 | Detritus | 329.4 | 174.7 | 392.4 | -- | -- | -- | -- | -- | -- |

B: biomass (g WW m^-2^); P/B: production/biomass (yr^-1^); Q/B: consumption/biomass (yr^-1^)
