# Supplementary material for: The Trophic Significance of the Indo-Pacific Humpback Dolphin, Sousa chinensis, in Western Taiwan
Source: PLoS One. 2016 Oct 25;11(10):e0165283. doi: 10.1371/journal.pone.0165283 (PMC5079652; doi:10.1371/journal.pone.0165283)
Supplement: S3 Table — (DOCX) [file pone.0165283.s003.docx]

**S3 Table. Diet (proportional) composition matrix for the compartments in the Dm model.**

|  | Prey \ predator | 1 | 2 | 3 | 4 | 5 | 6 | 7 | 8 | 9 | 10 | 11 | 12 | 13 | 14 | 15 | 16 | 17 |
| --- | --- | --- | --- | --- | --- | --- | --- | --- | --- | --- | --- | --- | --- | --- | --- | --- | --- | --- |
| 1 | Indo-Pacific humpback dolphins |  |  |  |  |  |  |  |  |  |  |  |  |  |  |  |  |  |
| 2 | Pelagic piscivorous fish | 0.02 | <0.01 |  |  |  |  |  |  |  |  |  |  |  |  |  |  |  |
| 3 | Benthic piscivorous fish |  |  | 0.09 |  |  |  |  |  |  |  |  |  |  |  |  |  |  |
| 4 | Large benthic-feeding fish | 0.60 | 0.01 | 0.01 | <0.01 |  |  |  |  |  |  |  |  |  |  |  |  |  |
| 5 | Small benthic-feeding fish | 0.38 |  | <0.01 | 0.06 |  |  |  | 0.01 | 0.01 |  |  |  |  |  |  |  |  |
| 6 | Zooplanktivorous fish | <0.01 | 0.02 |  |  |  |  |  | <0.01 |  |  |  |  |  |  |  |  |  |
| 7 | Omnivorous fish | <0.01 |  |  |  |  |  |  |  |  |  |  |  |  |  |  |  |  |
| 8 | Cephalopods |  | <0.01 | <0.01 | <0.01 |  |  |  | <0.01 |  |  |  |  |  |  |  |  |  |
| 9 | Stomatopods |  |  | 0.01 | 0.01 |  |  |  | <0.01 | <0.01 |  |  |  |  |  |  |  |  |
| 10 | Crabs |  |  | 0.08 | 0.05 | <0.01 |  |  |  | <0.01 |  |  |  |  |  |  |  |  |
| 11 | Shrimp |  | <0.01 | 0.20 | 0.20 | <0.01 |  | <0.01 | <0.01 | <0.01 | <0.01 |  |  |  |  |  |  |  |
| 12 | Gastropods |  |  | 0.03 | 0.05 | 0.01 |  | <0.01 |  | 0.03 | 0.01 | 0.01 |  |  |  |  |  |  |
| 13 | Bivalves |  |  | 0.13 | 0.15 | 0.03 |  | <0.01 |  | <0.01 | <0.01 |  | <0.01 |  |  |  |  |  |
| 14 | Amphipods |  |  | <0.01 | <0.01 | <0.01 | 0.02 | 0.01 |  | <0.01 | <0.01 |  | 0.01 |  |  |  |  |  |
| 15 | Polychaetes |  |  | 0.20 | 0.10 | 0.35 | <0.01 | <0.01 |  | 0.02 | 0.03 | 0.10 | 0.05 |  |  | <0.01 |  |  |
| 16 | Carnivorous zooplankton |  | 0.20 | 0.01 | 0.01 | 0.01 | 0.20 | 0.10 | 0.10 |  |  | 0.05 |  |  |  |  |  |  |
| 17 | Herbivorous zooplankton |  | 0.62 | 0.10 | 0.09 | 0.03 | 0.38 | 0.30 | 0.50 | 0.30 | 0.15 | 0.06 | 0.10 |  | 0.04 | 0.01 | 0.90 |  |
| 18 | Phytoplankton |  |  |  |  |  | 0.10 | 0.04 |  |  |  |  | 0.24 | 0.10 |  | 0.01 |  | 0.02 |
| 19 | Detritus |  | 0.15 | 0.15 | 0.28 | 0.57 | 0.30 | 0.54 | 0.39 | 0.64 | 0.81 | 0.79 | 0.60 | 0.90 | 0.96 | 0.98 | 0.10 | 0.98 |
