# Supplementary material for: The Trophic Significance of the Indo-Pacific Humpback Dolphin, Sousa chinensis, in Western Taiwan
Source: PLoS One. 2016 Oct 25;11(10):e0165283. doi: 10.1371/journal.pone.0165283 (PMC5079652; doi:10.1371/journal.pone.0165283)
Supplement: S5 Table — (DOCX) [file pone.0165283.s005.docx]

**S5 Table. Source of model input parameters.**

| Group name | B | P/B | Q/B | DC |
| --- | --- | --- | --- | --- |
| Indo-Pacific humpback dolphins | This study | Hoenig (1983) | Trites et al. (1997) | Ko (2011) |
| Pelagic piscivorous fish | This study | Fishbase | Fishbase | Duan et al. (2009) |
| Benthic piscivorous fish | This study | Fishbase | Fishbase | Duan et al. (2009) |
| Large benthic-feeding fish | This study | Fishbase | Fishbase | Duan et al. (2009) |
| Small benthic-feeding fish | This study | Fishbase | Fishbase | Duan et al. (2009) |
| Zooplanktivorous fish | This study | Fishbase | Fishbase | Duan et al. (2009) |
| Omnivorous fish | This study | Fishbase | Fishbase | Duan et al. (2009) |
| Cephalopods | This study | Brey (1995) | Pauly et al. (1993) | Opitz (1996) |
| Stomatopods | This study | Brey (1995) | Pauly et al. (1993) | Opitz (1996) |
| Crabs | This study | Brey (1995) | Lin et al. (1999) | Lin et al. (2006) |
| Shrimp | This study | Brey (1995) | Lin et al. (1999) | Lin et al. (2006) |
| Gastropods | This study | Brey (1995) | Riddle et al. (1990) | Opitz (1996) |
| Bivalves | This study | Brey (1995) | Riddle et al. (1990) | Opitz (1996) |
| Amphipods | EPA, Taiwan (2012, 2013)^a^ | Brey (1995) | Riddle et al. (1990) | Opitz (1996) |
| Polychaetes | EPA, Taiwan (2012, 2013)^a^ | Brey (1995) | Riddle et al. (1990) | Opitz (1996) |
| Carnivorous zooplankton | This study | Hirst and Bunker (2003) | Riddle et al. (1990) | Lin et al. (2006) |
| Herbivorous zooplankton | This study | Hirst et al. (2003) | Lin et al. (1999) | Lin et al. (2006) |
| Phytoplankton | This study | This study | -- | -- |
| Detritus | This study | -- | -- | -- |

B: biomass; P/B: production/biomass; Q/B: consumption/biomass; DC: diet composition

^a^ http://www.epa.gov.tw/np.asp?ctNode=32999&mp=epa
